# Profiling the *Trypanosoma cruzi* Phosphoproteome

**DOI:** 10.1371/journal.pone.0025381

**Published:** 2011-09-22

**Authors:** Fabricio K. Marchini, Lyris M. F. de Godoy, Rita C. P. Rampazzo, Daniela P. Pavoni, Christian M. Probst, Florian Gnad, Matthias Mann, Marco A. Krieger

**Affiliations:** 1 Instituto Carlos Chagas, Fiocruz, Curitiba, Paraná, Brazil; 2 Department of Proteomics and Signal Transduction, Max Planck Institute of Biochemistry, Martinsried, Germany; University of Texas-Houston Medical School, United States of America

## Abstract

Protein phosphorylation is a reversible post-translational modification essential for the regulation of several signal transduction pathways and biological processes in the living cell. Therefore, the identification of protein phosphorylation sites is crucial to understand cell signaling control at the molecular level. Based on mass spectrometry, recent studies have reported the large-scale mapping of phosphorylation sites in various eukaryotes and prokaryotes. However, little is known about the impact of phosphorylation in protozoan parasites. To in depth characterize the phosphoproteome of *Trypanosoma cruzi*, a parasite of the Kinetoplastida class, protein samples from cells at different phases of the metacyclogenesis – differentiation process of the parasites from non-infective epimastigotes to infective metacyclic trypomastigotes - were enriched for phosphopeptides using TiO_2_ chromatography and analyzed on an LTQ-Orbitrap mass spectrometer. In total, 1,671 proteins were identified, including 753 phosphoproteins, containing a total of 2,572 phosphorylation sites. The distribution of phosphorylated residues was 2,162 (84.1%) on serine, 384 (14.9%) on threonine and 26 (1.0%) on tyrosine. Here, we also report several consensus phosphorylation sequence motifs and as some of these conserved groups have enriched biological functions, we can infer the regulation by protein kinases of this functions. To our knowledge, our phosphoproteome is the most comprehensive dataset identified until now for Kinetoplastida species. Here we also were able to extract biological information and infer groups of sites phosphorylated by the same protein kinase. To make our data accessible to the scientific community, we uploaded our study to the data repositories PHOSIDA, Proteome Commons and TriTrypDB enabling researchers to access information about the phosphorylation sites identified here.

## Introduction

*T. cruzi* is the causal agent of Chagas disease, which has an infection prevalence estimated at 15 million cases [1], and affects neglected impoverished people primarily in Latin America, where it is mostly transmitted to humans by the triatomine insects. However, in the past decades, Chagas disease has been increasingly detected in the United States of America, Canada, many European and some Western Pacific countries, too. These occurrences are mainly caused by population mobility between Latin America and the rest of the world, infection through blood transfusion, vertical transmission (from infected mother to child) or organ donation (WHO 2010). The disease starts with an acute infection, which can be lethal in few cases, but usually evolves into a chronic stage that could lead to severe cardiopathy and ultimately to death. Once the infection takes place, the treatment is limited and the drugs currently available are highly toxic.

Four *T. cruzi* developmental stages have been well characterized, being two in the insect vector (epimastigotes and metacyclic trypomastigotes) and two in the mammalian hosts (amastigotes and bloodstream trypomastigotes) [2]. Epimastigotes and amastigotes are the non-infective, replicative forms, whereas metacyclic trypomastigotes and bloodstream trypomastigotes are the infective, non-replicative forms. Metacyclogenesis is the process by which epimastigotes differentiate into metacyclic trypomastigotes and acquire infectivity in the digestive tract of the insect vector. This process can be mimicked in vitro by cultivating epimastigotes under chemically defined conditions [3], making it possible to obtain intermediate differentiation forms as well as fully differentiated metacyclic trypomastigotes.

The adaptation to distinct environments (e.g. the insect's digestive tract and the interior of mammalian cells), with different temperatures, nutrient availability and immune response, requires major changes in the parasites' metabolism and gene expression. It is known that Kinetoplastida lack several transcriptional control mechanisms [4], [5], [6], giving post-transcriptional regulation a major role in controlling their gene expression [7], [8], [9], [10], [11]. In addition, there are also other dynamic control mechanisms, such as protein post-translational modifications, that still need to be better characterized in these organisms.

Protein phosphorylation is a reversible post-translational modification that constitutes a key mechanism to control protein function via their activation/inactivation or change in sub cellular localization. It regulates many physiological processes, including metabolic pathways, gene transcription, membrane transport, and cell division. Due to its importance in the control of signal transduction, the identification of protein phosphorylation sites is crucial to the understanding of cell signaling control at the molecular level. The relatively large number of protein kinase and phosphatase encoding genes and the importance of post-transcriptional control in protozoan parasites points to a fundamental role of protein phosphorylation in these unicellular organisms.

Different approaches have been applied to search for phosphorylated proteins on Kinetoplastida parasites. However, due to technical limitations, only few have been initially identified, including RNA polymerase II [12] and the Acidic ribosomal P proteins [13]. When it comes to identifying phosphorylation sites with traditional approaches, still just a few examples (e.g. Serine 12 on Histone H1 for *T. cruzi*) [14] were described for Kinetoplastida.

Recently, new sample preparation strategies [15], [16], [17], [18] and mass spectrometry-based methodologies [19], [20] have been developed to study protein phosphorylation, giving origin to the field of phosphoproteomics. Thus, thousands of phosphorylation sites can be identified, quantified and have their exact position determined in a given experiment, clearly opening new perspectives on cell signaling research. Despite these advances, however, only a few large-scale phosphoproteomic studies have been performed in Kinetoplastida so far, and the number of phosphoproteins and phosphorylation sites described in the literature is very limited. In a recent study Morales and co-workers identified 221 phosphorylated proteins on *Leishmania donovani*, using phosphoprotein enrichment and two-dimensional electrophoresis separation followed by tandem time-of-flight peptide sequencing [21]. With a combination of strong cation-exchange (SCX) fractionation, titanium dioxide (TiO_2_) chromatography phosphopeptide enrichment, and two different hybrid mass spectrometers, Nett and co-workers identified 491 phosphoproteins and 1,204 phosphorylation sites on bloodstream forms of *T. brucei* [22], Nett and co-workers also performed an elegant work direct to identify and localize tyrosines phosphorylated proteins [23]. Using a similar approach, Nakayasu and co-workers combined SCX fractionation, ion metal-affinity chromatography (IMAC) phosphopeptide enrichment on a low-resolution mass spectrometer to identify 119 phosphorylated proteins and a total of 192 unambiguous phosphorylation sites on epimastigote forms of *T. cruzi* [24].

Here, to obtain a more comprehensive picture of the *T. cruzi* phosphoproteome, we analyzed protein samples from parasites at five different moments of differentiation during the process of metacyclogenesis. The samples were enriched for phosphopeptides applying TiO_2_ chromatography, analyzed by high-accuracy mass spectrometry on a hybrid linear ion trap quadrupole–Orbitrap instrument (LTQ-Orbitrap, Thermo Scientific), and advanced bioinformatic tools were used to achieve unambiguous identification and site-specific localization of protein phosphorylation sites [25], [26]. We report the identification of several hundreds of phosphorylated proteins and more than two thousand phosphorylation sites.

Additional bioinformatic analyses performed found structural constraints and evolutionary conservation of the phosphorylation sites reported here. In addition, insights into biological processes regulated by phosphorylation could be inferred by the phosphorylation enrichment of proteins involved on cytoskeleton dynamic and protein kinase activity. Our study represents the most comprehensive phosphoproteomics dataset available for any of the Kinetoplastida parasites so far.

## Results and Discussion

### Profiling the Trypanosoma cruzi phosphoproteome

To increase the coverage of the phosphoproteome, we analyzed cells from parasites at five different time points during the differentiation process through which the parasite acquires infectivity (metacyclogenesis). To enhance the sampling of phosphorylated peptides we performed three sequential TiO_2_ enrichment steps for each sample. For better fragmentation of the phosphopeptides, MS/MS were acquired using multi-stage activation (MSA), also known as “pseudo-MS3” [20]. This strategy was repeated in the same way for 3 biological replicates, except for the fact that in the first biological replicate one of the time points (adhered 12 hours) was not present, resulting in a total of 42 LC-MS/MS runs for the complete experiment. The identification of phosphoproteins and phosphosites was based on the MaxQuant software [26].

Altogether, 130,459 MS/MS spectra were identified, corresponding to 5,513 non-redundant peptide sequences. The high-resolution data from the LTQ-Orbitrap mass spectrometer, combined with innovative computational strategies (MaxQuant), resulted in very high peptide mass accuracy for the precursor ions (average absolute mass deviation of 362 p.p.b.), which contributed considerably to the number and reliability of the identifications. A total of 1,671 protein groups were identified (Table S1), among which 753 were phosphorylated. For those phosphoproteins, 1,494 non-redundant phosphorylated peptides were determined. Some of the phosphopeptides were detected in more than one phosphorylation state (e.g. one, two, three phospho-groups) and, after removing this redundancy, 2,572 distinct phosphorylation sites presented a PTM score higher than 0.75 and a delta score higher than 5 (Table S2), indicating that their position could be determined with high confidence These phosphosites were used for the subsequent analysis.

To estimate the contribution of our data to the literature, we compared our phosphorylation sites with those previously described. To our knowledge, there has been only one phosphoproteomic study describing site-specific phosphorylation events for *T. cruzi*, performed by Nakayasu and co-workers, which analyzes only epimastigotes forms of the parasite and describes 192 unambiguous phosphorylation sites on 119 phosphorylated proteins [24]. For a direct comparison with this data, we considered only the phosphorylation sites detected in our study for the epimastigote form of *T. cruzi* and found that 1,511 phosphorylation sites were novel. Overall, 2,523 sites were new, resulting on a total of 2,715 phosphorylation sites described for *T. cruzi*. This represents an increase of more than 13-fold in the total number of phosphosites currently available for *T. cruzi*, making the present work also the largest dataset generated for any of the Kinetoplastida parasites. In addition, this is the only phosphoproteomics study to sample different life forms of the parasite so far.

Although the number of sites identified in our work is higher than the one from the previous study, the overlap between the two datasets is relatively low. This could be explained by several experimental differences existing between the two studies, such as the use of different *T. cruzi* strains, phosphopeptide enrichment approaches [18], mass spectrometry platforms and validation criteria for identification and localization of the phosphorylation sites.

### Structural constraints and conservation of phosphorylation sites

We derived secondary structure and accessibility constraints to phosphorylation sites using the prediction method SABLE 2.0 [27]. SABLE assigns the predicted secondary structure (helix, β-sheet, or turn/loop) to each residue of a given protein sequence. In addition, it calculates accessibility values ranging from 0 (fully buried) to 9 (fully exposed). As in other eukaryotic phosphoproteomes [25], [28], phosphorylation sites are almost exclusively located in turn and loop regions of the proteins surface (Figure S1). In total, 83% of phosphorylated serines were predicted to be located in non-regularly structured regions in comparison to 67% of the serines that were not identified to be phosphorylated on the same proteins (p<10^−50^ based on Chi Square Statistics). In concordance, the predicted accessibilities were also significantly higher for phosphorylated residues compared to their non-phosphorylated counterpart (p<10^−23^ based on t statistics) (Figure S2). These structural constraints are essential for kinase/phosphatase substrate accessibility, as well as for subsequent functional and structural effects on the substrate protein.

Based on two-directional BLASTP applications [29], [30], we derived homologs of the whole *T. cruzi* proteome in all domains of life (Figure S3). Interestingly, the proportion of phosphorylated *T. cruzi* proteins that have homologs in eukaryotic species is significantly higher than the proportion of non-phosphorylated *T. cruzi* proteins that show homologous counterparts in other eukaryotic species. However, in the prokaryotic domain, phosphorylated *T. cruzi* proteins are as conserved as non-phosphorylated *T. cruzi* proteins.

### Distribution of phosphorylation sites

The distribution of phosphorylation events in number of sites per protein and peptide is illustrated in Figure S4. When considering the categories individually, although the highest number of phosphoproteins was found to have just one phosphorylation site, if the phosphoproteome is considered as a whole, the majority of phosphorylated proteins had multiple phosphorylation sites: 73% having more than two and 53% having three or more phosphorylation sites (Figure S4). Similarly, the majority of non-redundant phosphopeptide sequences contained multiple phosphorylation sites, despite the fact that the most prominent individual class corresponded to singly phosphorylated peptides. Likewise, most of the MS/MS spectra identified belonged to multiply phosphorylated peptides. The most frequently class identified (as estimated by spectra count) was the one of doubly phosphorylated peptides, which accounted for more than 35% of the total identified MS/MS (Figure S5), this could be a biological characteristic or technical conditions such as TiO_2_ enrichment.

The S/T/Y distribution of the phosphorylated sites identified by our work for *T. cruzi* mapped to 2,162 pS (84.1%)/384 pT (14.9%)/26 pY (1.0%) amino acid residues. Although dedicated tyrosine protein kinases (PKs) are still unknown, the occurrence of tyrosine phosphorylation has been well reported in trypanosomatids [23], [31]. In this work, a total of 25 proteins were revealed to be tyrosine phosphorylated, and 26 pY sites were exactly located (Table S2). Tyrosine phosphorylation is known to play a key role in cell signaling, and in our dataset different classes of proteins, such as calpain-like cysteine peptidase, leucine-rich repeat protein and several protein kinases were found to be phosphorylated on tyrosine residues. The group of protein kinases (further discussed below) is of particular interest, and accounted for more than 30% of the phosphotyrosine sites identified in our dataset.

### The kinome phosphoproteome

The completion of the genome sequence of the three model trypanosomatids, *T. brucei* [32], *T. cruzi* [33], [34] and *L. major* [35], made possible to exploit these data to better understand important protein groups of these parasites. Parsons and co-workers analyzed the components of the kinome and described 179, 156, and 171 eukaryotic protein kinases (ePKs) and 17, 20, 19 atypical PKs (aPKs) for *L. major*, *T. brucei* and *T. cruzi*, respectively [36]. The number of PKs for the trypanosomatids is considerably larger than that described for another intracellular parasite, *Plasmodium falciparum*, which possesses 65 ePKs and 20 ePK-related sequences [37]. In addition, for some PK families the gene number is proportionally higher than *Homo sapiens* [38].

Despite the fact that a kinase-selective enrichment approach [39] was not used, 42 phosphorylated protein kinases were identified in our analysis, corresponding to a total of 125 phosphorylation sites (Table S3). Considering that *T. cruzi* contains 171 ePK/19 aPK in total [36], our dataset shows evidence of phosphorylation for about 22% of the kinome, covering different kinase families (Table 1).

**Table 1:** Phosphorylated protein kinases.

ePK/aPK	Family	Sub-Family	Members	Phosphoproteins
**ePK**	**AGC**	-	7	1
		NDR	1	0
		PKA	3	2
		RSK	1	0
		**TOTAL**	12	3
	**CAMK**	-	7	0
		CAMKL	6	2
		**TOTAL**	13	2
	**CK1**	CK1	7	1
		TTBK	1	0
		**TOTAL**	8	1
	**CMGC**	-	1	0
		CDK	10	1
		CLK	5	0
		DYRK	7	2
		GSK	2	1
		CDKL (MAPK-like)	1	1
		MAPK	11	5
		RCK (MAPK-like)	3	2
		SRPK	2	1
		**TOTAL**	42	13
	**Other**	AUR	3	0
		CAMKK	4	1
		CK2	2	0
		NAK	1	0
		NEK	22	7
		PEK	2	1
		PLK	2	0
		TLK	1	0
		ULK	1	1
		VPS	1	0
		WEE	2	0
		**TOTAL**	42	10
	**STE**	-	8	0
		STE7	2	0
		STE11	18	4
		STE20	3	0
		**TOTAL**	31	4
	**Unique**	**TOTAL**	23	6
	**TOTAL**		**171**	**39**
**aPK**	**A6**	**TOTAL**	1	0
	**ABC1**	**TOTAL**	5	0
	**alpha**	**TOTAL**	1	0
	**Bud32**	**TOTAL**	1	0
	**PDK**	**TOTAL**	3	0
	**PIKK**	ATM	1	0
		ATR	1	0
		FRAP	4	2
		RIO1	1	0
		RIO2	1	1
		**TOTAL**	8	3
	**TOTAL**		**19**	**3**

Members, number of protein kinase genes identified in the *T. cruzi* genome; Phosphoproteins, number of protein kinases identified in the phosproteomic data.

Another interesting consideration is that, as mentioned above, more than 30% of the phosphorylated tyrosines detected were found on protein kinases. In addition, the *T. cruzi* kinome has the S/T/Y phosphorylation sites distribution shifted towards the phosphotyrosines (6% of all the phosphorylation sites found on PKs are pYs), showing a 6-fold enrichment compared to the rate found for the total phosphoproteome.

Parsons and colleagues [36] propose that the tyrosine phosphorylation activity in trypanosomatids is likely to be due to the action of atypical tyrosine kinases such as Wee1 and dual-specificity tyrosine phosphorylation–regulated kinases (DYRKs), that can also phosphorylate serine/threonine and tyrosine residues. At the cellular level, they have been described as regulators of differentiation, cell cycle progression, and apoptosis. All DYRKs autophosphorylate a critical tyrosine in their activation loop, but phosphorylate their substrates on serines or threonines, which could be the explanation for the phosphorylated tyrosine of the *Trypanosoma cruzi* DYRK (Tc00.1047053511249.60). This could also be an example of autophosphorylation events found in *T. cruzi*.

**Figure 1:**
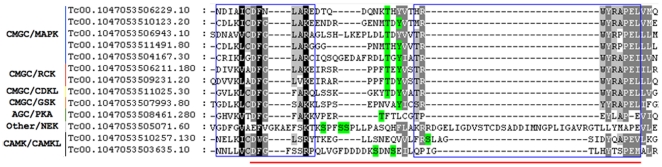
Kinases with phosphorylated activation loop. Blue boxes represent the conserved regions. Red line at the bottom represents the activation region, with the non-organized activation loop in between the two conserved regions. The identified phosphorylation sites are highlighted in green.

Phosphorylation in the activation loop segment, which represents a conserved structural element within the protein kinase domain, has been implicated in the stabilization of the catalytically active state of many eukaryotic protein kinases [40]. Phosphorylation at the activation loop was identified in 13 protein kinases, belonging to seven different families: (i) five mitogen-activated protein kinases (MAPKs); (ii) two RCKs and (iii) one CDKL - both families similar to MAPKs; (iv) two CAMKL; (v) one PKA; (vi) one NEK and (vii) one glycogen synthase protein kinase (GSK) (Figure 1). Therefore, the presence of phosphorylation on the activation loop, described here, could be a sign of activity regulation of these kinases. The glycogen synthase kinases are constitutively phosphorylated on a tyrosine residue in their activation loop, which is required for their full enzymatic activity. As previously described [24], for GSK we also identify tyrosine 187 phosphorylated, which is located, at its activation loop, suggesting its auto-activation. In addition, we found evidence of activation for 6 of the 15 described *T. cruzi* MAPK or MAPK-like kinases, as demonstrated by the concomitant presence of both tyrosine and threonine phosphorylation on their activation loop, similar to what was previously described for *T. brucei* [22].

Although the identification of protein kinases responsible for tyrosine phosphorylation in TriTryps is still missing, the identification of phosphorylated tyrosines at the activation loop of protein kinases reveals some phosphorylated tyrosine regulated PKs.

### Functional characterization of the T. cruzi phosphoproteome

Intending to determine the biological processes correlating genes with Gene Ontology (GO) terms that share common biological aspects affected/regulated by protein phosphorylation during the *T. cruzi* metacyclogenesis, the phosphorylated proteins were submitted to GO enrichment analysis compared to the MCL *T. cruzi* gene set (see Material and methods section), to search for differential GO representation (Table 2).

**Table 2:** GO enrichment analysis of phosphorylated proteins.

GO Term	GO Name	FDR	# Ph	# Genome	X
GO:0004672	protein kinase activity	2.06E-07	42	298	3.17
GO:0006464	protein modification process	1.26E-05	59	609	2.21
GO:0005509	calcium ion binding	4.36E-05	16	75	4.50
GO:0006519	amino acid and derivative metabolic process	5.05E-04	45	488	2.03
GO:0000166	nucleotide binding	1.04E-02	85	1258	1.52
GO:0005856	Cytoskeleton	1.63E-02	23	237	2.04
GO:0007165	signal transduction	2.67E-02	23	249	1.94
GO:0005929	Cilium	3.02E-02	5	20	5.11

FDR, False Discovery Rate; # Ph, number of phosphorylated proteins; # Genome, number of proteins present in the genome; X, enrichment of each GO term.

Eight functional groups were overrepresented at the phosphorylated proteins compared to the genome. Interestingly, some members of this group are related to the major morphological changes correlated with modifications that happen during the metacyclogenesis, since there is a change at the cell shape and at the positioning of cellular organelles during the parasite's differentiation. Finding terms as cytoskeleton and cilium reinforces the importance of this approach to identify key regulators of biological processes. The enrichment of both terms is supported by the localization of phosphorylated proteins at the flagellum [23], as well as protein kinases related to roles in the movement of organelles and cell division [41], [42], [43].

The protein kinases are effectors of signal transduction pathways and can also be regulated by phosphorylation. The discovery of “protein kinase activity” and “signal transduction” GO terms that were overrepresented at phosphorylated proteins supports this idea. As the PKs trigger a wide range of processes, they have to be tightly regulated. Moreover, the members of the kinome also regulate each other's function trough phosphorylation. For example, some kinases contain specific phosphorylation sites within the activation loop that are important for determining the conformation of the loop and, consequently, the activity of the phosphorylated kinase [40]. This result is reinforced by the identification of phosphorylation at the activation loop of several protein kinases. Both evidences demonstrate the phosphorylation control of this key regulatory function. The other overrepresented GO terms found (Amino acid and derivative metabolic process; Protein modification process; Nucleotide binding; Calcium ion binding) are good candidates for better characterization of key processes regulated by phosphorylation during this cellular differentiation.

### Phosphorylation Motifs

The number of phosphorylation sites revealed now for trypanosomes can assist classical characterization of proteins and pathways. Even though there is still a lack of information to correlate the phosphorylation sites to protein kinases, here we group similar phosphorylation sites that could be phosphorylated by a specific protein kinase or few protein kinases that share the same amino acids specificity at their substrates. This results on groups of phosphorylation sites that regulate similar biological functions being phosphorylated by a protein kinase that has affinity by the motif present on these sites. To initially address the issue of which kinases are the effectors of the identified phosphorylation sites, an approach that could give us motifs of phosphorylation sites was used. Using Motif-X [44], The conservation of the amino acids close to the phospho amino acid was used to extract patterns from our results, revealing phosphorylation motifs.

**Figure 2:**
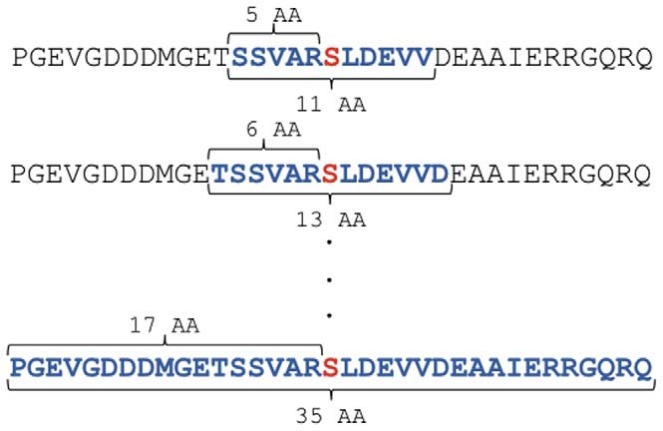
Sequence windows used to extract phosphorylation motifs. The sequence represents an example of the phosphorylated region of a protein, showing the backbone structure of the different window sizes used to extract phosphorylation motifs. The red letter represents the phosphorylated residue (always centered), the blue letters were emphasized to show the surrounding amino acids included in each of the window sizes.

Using 13 different window size sets of data (from 11 to 35 amino acids) (Figure 2) for both S/T phosphorylation sites, 248 motifs have been identified (Table S4). Although redundancy is apparent, redundant motifs are formed not only by a similar core of sites but also by different ones, as each window size presents different situations of motif extraction.

Despite the absence of a direct link between the substrate and the protein kinase we could group the phosphorylation sites based on similar amino acid sequences surrounding the phospho amino acid. Twenty-four different motifs were found using 19 amino acids as window size, the bigger motif set found.

The results obtained from the enrichment analysis points to the regulation of specific biological functions by protein kinases. To map the predominant functional themes of each motif in order to search for a similar biological regulation, we used statistical analysis to test the overrepresentation of GO terms in the different sets of motifs containing proteins present compared to the population set (GO annotated *T. cruzi* MCL), through the Blast2GO software [45].

**Figure 3:**
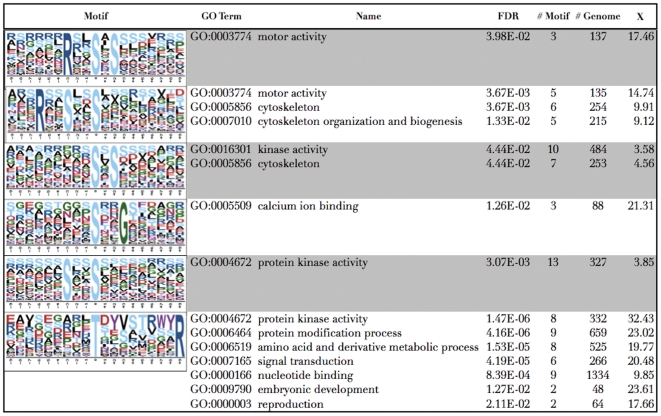
Motifs found to have GO terms overrepresented.

This result can give some clues about the group of phosphorylated proteins found with meaningful related motifs, as this represents a group of proteins with similar biological properties regulated by a Protein kinase. Here we show the GO enrichment of six phosphorylation motifs (Figure 3): 1) RxxpSxS (motor activity), 2) RxxSxxpS (motor activity, cytoskeleton and cytoskeleton organization and biogenesis), 3) pSxS (kinase activity and cytoskeleton), 4) pSxxG (calcium ion binding), 5) SxxpS (protein kinase activity) and 6) pTxxxxxxxxR (protein kinase activity, protein modification process, amino acid and derivative metabolic process, signal transduction, nucleotide binding, embryonic development and reproduction). These motifs (and the proteins correlated) can infer key regulated proteins for further characterizations.

Thus, this work can greatly contribute to improve the knowledge about trypanosomatids' biology, helping to understand key regulatory signal transduction events at the molecular level. Even though this work gave interesting information and greatly increased the number of phosphorylation sites known, further quantitative and protein kinase-related substrate approaches are still needed to clarify the regulated pathways more dynamically to help understand the complex biological puzzle.

### Phosphoproteome dynamics during T. cruzi metacyclogenesis

The phosphorylation dataset described and characterized here was obtained from 5 time points during the *T. cruzi* metacyclogenesis differentiation process. To start digging the proteins that may be regulated during cellular differentiation, we chose to look for processes/functions/components (GO terms) overrepresented at each time point (Table 3).

**Table 3:** GO enrichment analysis of phosphorylated proteins across metacyclogenesis.

GO Term	Name	Type	FDR (<0.05)	# Ph	# Genome	X
Epimastigostes						
GO:0005509	calcium ion binding	F	1.40E-02	10	81	4.00
GO:0004672	protein kinase activity	F	1.40E-02	23	317	2.44
GO:0006464	protein modification process	P	3.60E-02	35	633	1.89
Stress 30 minutes						
GO:0003824	calcium ion binding	F	9.00E-03	10	81	4.62
Stress 2 hours						
GO:0004672	protein kinase activity	F	1.60E-02	21	319	2.73
GO:0005509	calcium ion binding	F	4.80E-02	8	83	3.79
Adhered 12 hours						
GO:0005856	Cytoskeleton	C	2.30E-03	18	242	3.24
GO:0004672	protein kinase activity	F	5.90E-05	25	315	3.62
GO:0005509	calcium ion binding	F	6.40E-03	9	82	4.59
GO:0000166	nucleotide binding	F	7.90E-03	51	1292	1.89
GO:0003774	motor activity	F	2.30E-02	10	130	3.21
GO:0006464	protein modification process	P	6.60E-03	31	637	2.20
Metacyclic						
GO:0005856	Cytoskeleton	C	9.00E-04	21	239	3.00
GO:0004672	protein kinase activity	F	4.10E-05	29	311	3.31
GO:0005509	calcium ion binding	F	5.10E-04	12	79	5.05
GO:0000166	nucleotide binding	F	1.60E-02	60	1283	1.71
GO:0003774	motor activity	F	3.20E-02	11	129	2.79
GO:0006464	protein modification process	P	4.30E-03	37	631	2.07

F, molecular function; C, cellular component; P, biological process; FDR, False Discovery Rate; # Ph, number of phosphorylated proteins ; # Genome, number of proteins present in the genome; X, enrichment of each GO term.

All the time points showed enrichment for calcium ion binding, protein kinase activity and protein modification process, meaning that the proteins involved in all these functions/processes are constantly being phosphorylated. Towards the late phases (Ad12h and Meta), however, three new biological functions arised as overrepresented: Cytoskeleton, motor activity and nucleotide binding. The regulation of cytoskeleton and motor activity by protein phosphorylation, specifically at the end of metacyclogenesis, could indicate the key regulated molecules responsible for the morphological changes that give origin to metacyclic trypomastigotes. The nucleotide binding function, although too general, could indicate that RNA binding proteins and cyclic nucleotide binding proteins are also being regulated at the late stages of differentiation.

Despite the fact that the data is not really quantitative, as our experimental design is a time point situation across *T. cruzi* differentiation, it was interesting to observe similar processes being regulated in a timely manner, conferring certain robustness to these conclusions, since the global result would be taken into account and not just each time point isolated.

## Materials and Methods

### Cells

*Trypanosoma cruzi* Dm28c [46] epimastigotes were cultured in liver infusion tryptose (LIT) medium [47] supplemented with 10% fetal bovine serum without agitation at 28°C. For in vitro differentiation, epimastigotes were harvested from LIT medium after 5 days of culture, by centrifugation at 7,000 g for 5 min at 20°C. They were incubated for 2 h at 28°C in TAU medium (190 mM NaCl, 17 mM KCl, 2 mM MgCl2, 2 mM CaCl2, 8 mM phosphate buffer pH 6.0) at a density of 5×108 parasites/ml and then diluted 1∶100 in TAU3AAG medium (TAU supplemented with 10 mM l-proline, 50 mM l-sodium glutamate, 2 mM l-sodium aspartate, and 10 mM d-glucose) in tissue culture flasks not exceeding 1 cm height of medium. Metacyclic trypomastigotes were purified from cell culture supernatants by DEAE-cellulose chromatography after 72 h of incubation at 28°C [48].

*T. cruzi* epimastigotes were grown in vitro until they reached early stationary phase, stimulated to undergo metacyclogenesis and collected at 5 time points during the process of differentiation: epimastigotes (Epi); epimastigotes submitted to nutritional stress for 30 minutes (St30m); cells submitted to nutritional stress for 2 hours (St2h); cells allowed to adhere for 12 hours (Ad12h) and metacyclic trypomastigotes (Meta).

### Protein extraction

Cells (5×10^8^ epimastigotes forms and 1×10^9^ metacyclic trypomastigotes) were washed three times in phosphate buffer saline, pH 7.2 (8,000 g for 10 minutes at room temperature), resuspended in 400 µL lysis buffer (7 M Urea, 2 M Thiourea, 1 mM DTT, 1% n-octylglucoside, 50 mM sodium fluoride, 10 mM sodium pyrophosphate, 1 mM sodium orthovanadate, 10 mM β-glicerophosphate, protease inhibitors (Amersham), nuclease mix (Amersham)) and incubated with agitation for 30 minutes. The debris was removed by centrifugation and the supernatants were collected and stored at −70°C until use.

### Protein digestion

For in-solution digestion, protein extracts were reduced with 1 mM dithiothreitol, alkylated with 5.5 mM iodoacetamide, digested for three hours with endoproteinase Lys-C (Waco), diluted four times with 20 mM ammonium hydrogen carbonate and further digested overnight with trypsin (Promega). A protease/protein ratio of 1/50 was used for both enzymes. The peptide digests were then submitted to TiO_2_ chromatography for phosphopeptide enrichment and analyzed by LC-MS/MS as described below.

### TiO_2_ enrichment of phosphopeptides

Peptide digests were submitted to batch-wise enrichment of phosphopeptides using TiO_2_ chromatography, as previously described [49], [50]. Briefly, TiO_2_ beads (GL Sciences) and samples were pre-incubated with 2,5-dihydroxybenzoic acid (5 mg/mL final concentration) in 80% acetonitrile. In each cycle of phosphopeptide enrichment, TiO_2_ beads (approx. 1 mg) were added to the samples and incubated for 30 minutes at room temperature, shaking at 1,000 rpm. After incubation, the beads were spun down, washed 2 times with 50% acetonitrile, 0.2% trifluoroacetic acid in water, resuspended in washing buffer, loaded onto a RP-C8 Stage-tip column and washed one more time. Bound peptides were eluted from the column with a 40% acetonitrile, 0.5% ammonium solution, pH>10.5, concentrated to approximately 10 µl, reconstituted in 100 µl of 2% acetonitrile, 1% TFA in water, desalted using RP-C18 StageTip [51] columns and analyzed by mass spectrometry.

### NanoLC-MS/MS analysis

Peptide mixtures were separated by online reversed-phase (RP) nanoscale capillary liquid chromatography (nanoLC) and analyzed by electrospray tandem mass spectrometry (ES MS/MS). The experiments were performed with an Agilent 1100 nanoflow system connected to an LTQ-Orbitrap mass spectrometer (Thermo) equipped with a nanoelectrospray ion source (Proxeon Biosystems). Binding and chromatographic separation of the peptides took place in a 15 cm fused silica emitter (75 µm inner diameter) in-house packed with reversed-phase ReproSil-Pur C18-AQ 3 µm resin (Dr. Maisch GmbH, Ammerbuch-Entringen, Germany). Peptide mixtures were injected onto the column with a flow of 500 nL/min and subsequently eluted with a flow of 250 nL/min from 5% to 40% MeCN in 0.5% acetic acid, in an 80 or 120 min gradient. The mass spectrometer was operated in data dependent mode to automatically switch between MS and MS/MS (MS2) acquisition. Survey full scan MS spectra (from m/z 300–1600) were acquired in the Orbitrap analyzer with resolution R = 60,000 at m/z 400 (after accumulation to a target value of 1,000,000 in the linear ion trap). The 5–10 most intense ions were sequentially isolated and fragmented in the linear ion trap using collisionally induced dissociation at a target value of 10,000. Former target ions selected for MS/MS were dynamically excluded for 30 seconds. Total cycle time was of approximately three seconds. The general mass spectrometric conditions were: spray voltage, 2.4 kV; no sheath and auxiliary gas flow; ion transfer tube temperature, 100°C; collision gas pressure, 1.3 mTorr; normalized collision energy using wide-band activation mode; 35% for MS2. Ion selection thresholds were: 250 counts for MS2. An activation q = 0.25 and activation time of 30 ms was applied in MS2 acquisitions. Multistage activation was enabled in all MS/MS events to improve fragmentation spectra of phosphopeptides and the “lock mass” option was enabled in all full scans to improve mass accuracy of precursor ions.

### Data processing and validation

Proteins were identified by automated database searching (Mascot Daemon, Matrix Science) against a *T. cruzi* protein sequence database, containing 19,615 protein sequences downloaded from GeneDB, http://www.genedb.org/) and a ‘decoy database’, prepared by sequence reversing each entry of the amino acid sequence from the genome annotation. This database was complemented with frequently observed contaminants (porcine trypsin, Achromobacter lyticus lysyl endopeptidase and human keratins) and their reversed sequences as well. Search parameters specified a MS tolerance of 5 ppm, a MS/MS tolerance at 0.5 Da and full trypsin specificity, allowing for up to three missed cleavages. Carbamidomethylation of cysteine was set as a fixed modification and oxidation of methionines, N-terminal protein acetylation and N-pyroglutamate were allowed as variable modifications. The peak list generation for Mascot searches, as well as protein validation, protein grouping and phosphosite localization, were done using MaxQuant [26]. The FDRs were calculated based on the number of reverse hits from the searches against the decoy database. Peptides were required to have at least 6 amino acids in length, and a FDR of 0.01 was applied at the levels of peptides, proteins and phosphorylation sites. As described previously, a separate FDR calculation is necessary for substoichiometric modifications (e.g. phosphotyrosines), decreasing false positives and therefore avoiding overestimating the occurrence of such modifications in the sample [28]. This calculation is incorporated at MaxQuant platform. Only the phosphorylation sites PTM with a score higher than 0.75 and a delta score higher than 5 were considered. The comparison against the data described here and the publication from Nakayasu was done using only the phosphorylation sites that have been localized by that paper for just one position.

To make our data accessible to the scientific community, we uploaded our study to the data repositories PHOSIDA [52] (http://www.phosida.org), Proteome Commons (https://www.proteomecommons.org) and TriTrypDB (http://www.tritrypdb.org), enabling researchers to access information about the phosphorylation sites identified here.

### Motif extraction

With the exact localization of serine/threonine phosphorylation sites, the Motif-X algorithm [44] was used to extract phosphorylation motifs. As input data to Motif-X, files containing S/T with 11 to 35 amino acids window for each phosphorylation position were created. The set up used was based on 1E-6 stringency value, 20 occurrences, with the size adjusted for each sequence window set, centralizing the specific phosphorylated amino acid. To be used as background for the statistical calculations, the redundancy of the *T. cruzi* genome had to be analyzed. A file containing 19,615 protein sequence entries resulted in 13,525 entries, which were derived from a graphic clusterization by flow simulation using the algorithm MCL with an inflation of 1.835. The tyrosine phosphorylated sites were not used for this analysis since the number of identified sites is similar to the occurrences.

### GO enrichment analysis

To perform statistical analysis for overrepresentation of Gene Ontology (GO) terms on the test sets we used the Blat2GO [45], using the Fisher's test with an FDR of 0.05 and looking for the most specific terms.

## Supporting Information

Figure S1 **Proportion of residues located in loops.** Proportion of phosphorylated (red) and non-phosphorylated (blue) serines, threonines and tyrosines that are located in loops, according to secondary structure prediction. The phosphorylated residues are significantly higher localized in loops and turns when compared with their non-phosphorylated counterparts.
(TIFF)

Figure S2 **S/T/Y average accessibility.** Average accessibility of phosphorylated (red) and non-phosphorylated (blue) serines and threonines according to secondary structure prediction. The predicted average accessibilities of *T. cruzi* phosphorylation sites were found to be significantly higher than the accessibilities of non-phosphorylated sites.
(TIFF)

Figure S3 **Phosphoproteome similarity on all domains of life.** Homologs of all *T. cruzi* proteins were derived and the conservation of non-phosphorylated (blue) and phosphorylated (red) proteins were accessed across a range of both prokaryotic and eukaryotic organisms. When compared to other eukaryotic species, the percentage of *T. cruzi* phosphorylated proteins that have homologs is significantly higher than that of the non-phosphorylated proteins. On the other hand, in the prokaryotic domain, phosphorylated *T. cruzi* proteins are as conserved as the non-phosphorylated ones.
(TIFF)

Figure S4 **Protein phosphorylation distribution.** Proteins were binned according to their number of phosphorylation sites, as determined by LC-MS/MS. Bars represent the number of proteins belonging to each category bin (red) and the summed number of phosphorylation sites derived from the proteins in each respective class (blue).
(TIFF)

Figure S5 **Peptide phosphorylation distribution.** Peptides were binned according to their number of phosphorylation sites, as determined by LC-MS/MS. Bars represent the number of non-redundant phosphorylated peptides belonging to each category bin (red) and the total number of MS/MS spectra identifying the phosphopeptides of the corresponding bin (blue).
(TIFF)

Table S1 **Proteins identified.** Proteins identified and ordered by phosphorylation presence.
(XLS)

Table S2 **Phosphorylation sites.** Table containing description for each phosphorylation site identified. The informations are: IDs for each protein group; proteins descriptions; position of the phosphorylation at each protein; phosphorylated amino acid; localization probabilities for each site; scores for the identification; best protein kinase motif and its match probability; sequences with probabilities and scores; for each site; peptide charge; mass to charge; presence (“+”) or absence (“ ”) at the *Trypanosoma cruzi* time points.
(XLS)

Table S3 **Phosphorylated protein kinases.** Description of protein kinases found phosphorylated.
(XLS)

Table S4 **Description of phosphorylation site motifs.** Detailed stratification of the numbers of phosphorylation sites grouped at each window size and motif.
(XLS)

## References

[1] Coura JR, Dias JC (2009). Epidemiology, control and surveillance of Chagas disease: 100 years after its discovery.. Mem Inst Oswaldo Cruz.

[2] De Souza W (2002). Basic cell biology of Trypanosoma cruzi.. Curr Pharm Des.

[3] Contreras VT, Salles JM, Thomas N, Morel CM, Goldenberg S (1985). In vitro differentiation of Trypanosoma cruzi under chemically defined conditions.. Mol Biochem Parasitol.

[4] Teixeira SM, Kirchhoff LV, Donelson JE (1995). Post-transcriptional elements regulating expression of mRNAs from the amastin/tuzin gene cluster of Trypanosoma cruzi.. J Biol Chem.

[5] Di Noia JM, D'Orso I, Sanchez DO, Frasch AC (2000). AU-rich elements in the 3′-untranslated region of a new mucin-type gene family of Trypanosoma cruzi confers mRNA instability and modulates translation efficiency.. J Biol Chem.

[6] Clayton C, Shapira M (2007). Post-transcriptional regulation of gene expression in trypanosomes and leishmanias.. Mol Biochem Parasitol.

[7] D'Orso I, De Gaudenzi JG, Frasch AC (2003). RNA-binding proteins and mRNA turnover in trypanosomes.. Trends Parasitol.

[8] Batista JA, Teixeira SM, Donelson JE, Kirchhoff LV, de Sa CM (1994). Characterization of a Trypanosoma cruzi poly(A)-binding protein and its genes.. Mol Biochem Parasitol.

[9] Xu P, Wen L, Benegal G, Wang X, Buck GA (2001). Identification of a spliced leader RNA binding protein from Trypanosoma cruzi.. Mol Biochem Parasitol.

[10] Dallagiovanna B, Perez L, Sotelo-Silveira J, Smircich P, Duhagon MA (2005). Trypanosoma cruzi: molecular characterization of TcPUF6, a Pumilio protein.. Exp Parasitol.

[11] Espinosa JM, Portal D, Lobo GS, Pereira CA, Alonso GD (2003). Trypanosoma cruzi poly-zinc finger protein: a novel DNA/RNA-binding CCHC-zinc finger protein.. Mol Biochem Parasitol.

[12] Chapman AB, Agabian N (1994). Trypanosoma brucei RNA polymerase II is phosphorylated in the absence of carboxyl-terminal domain heptapeptide repeats.. J Biol Chem.

[13] Gomez EB, Medina G, Ballesta JP, Levin MJ, Tellez-Inon MT (2001). Acidic ribosomal P proteins are phosphorylated in Trypanosoma cruzi.. Int J Parasitol.

[14] da Cunha JP, Nakayasu ES, Elias MC, Pimenta DC, Tellez-Inon MT (2005). Trypanosoma cruzi histone H1 is phosphorylated in a typical cyclin dependent kinase site accordingly to the cell cycle.. Mol Biochem Parasitol.

[15] Scanff P, Yvon M, Pelissier JP (1991). Immobilized Fe3+ affinity chromatographic isolation of phosphopeptides.. J Chromatogr.

[16] Posewitz MC, Tempst P (1999). Immobilized gallium(III) affinity chromatography of phosphopeptides.. Anal Chem.

[17] Thingholm TE, Jorgensen TJ, Jensen ON, Larsen MR (2006). Highly selective enrichment of phosphorylated peptides using titanium dioxide.. Nat Protoc.

[18] Bodenmiller B, Mueller LN, Mueller M, Domon B, Aebersold R (2007). Reproducible isolation of distinct, overlapping segments of the phosphoproteome.. Nat Methods.

[19] Syka JE, Coon JJ, Schroeder MJ, Shabanowitz J, Hunt DF (2004). Peptide and protein sequence analysis by electron transfer dissociation mass spectrometry.. Proc Natl Acad Sci U S A.

[20] Schroeder MJ, Shabanowitz J, Schwartz JC, Hunt DF, Coon JJ (2004). A neutral loss activation method for improved phosphopeptide sequence analysis by quadrupole ion trap mass spectrometry.. Anal Chem.

[21] Morales MA, Watanabe R, Laurent C, Lenormand P, Rousselle JC (2008). Phosphoproteomic analysis of Leishmania donovani pro- and amastigote stages.. Proteomics.

[22] Nett IR, Martin DM, Miranda-Saavedra D, Lamont D, Barber JD (2009). The phosphoproteome of bloodstream form Trypanosoma brucei, causative agent of African sleeping sickness.. Mol Cell Proteomics.

[23] Nett IR, Davidson L, Lamont D, Ferguson MA (2009). Identification and specific localization of tyrosine-phosphorylated proteins in Trypanosoma brucei.. Eukaryot Cell.

[24] Nakayasu ES, Gaynor MR, Sobreira TJ, Ross JA, Almeida IC (2009). Phosphoproteomic analysis of the human pathogen Trypanosoma cruzi at the epimastigote stage.. Proteomics.

[25] Olsen JV, Blagoev B, Gnad F, Macek B, Kumar C (2006). Global, in vivo, and site-specific phosphorylation dynamics in signaling networks.. Cell.

[26] Cox J, Mann M (2008). MaxQuant enables high peptide identification rates, individualized p.p.b.-range mass accuracies and proteome-wide protein quantification.. Nat Biotechnol.

[27] Adamczak R, Porollo A, Meller J (2005). Combining prediction of secondary structure and solvent accessibility in proteins.. Proteins.

[28] Gnad F, de Godoy LM, Cox J, Neuhauser N, Ren S (2009). High-accuracy identification and bioinformatic analysis of in vivo protein phosphorylation sites in yeast.. Proteomics.

[29] Altschul SF, Gish W, Miller W, Myers EW, Lipman DJ (1990). Basic local alignment search tool.. J Mol Biol.

[30] O'Brien KP, Remm M, Sonnhammer EL (2005). Inparanoid: a comprehensive database of eukaryotic orthologs.. Nucleic Acids Res.

[31] Parsons M, Valentine M, Deans J, Schieven GL, Ledbetter JA (1991). Distinct patterns of tyrosine phosphorylation during the life cycle of Trypanosoma brucei.. Mol Biochem Parasitol.

[32] Berriman M, Ghedin E, Hertz-Fowler C, Blandin G, Renauld H (2005). The genome of the African trypanosome Trypanosoma brucei.. Science.

[33] El-Sayed NM, Myler PJ, Bartholomeu DC, Nilsson D, Aggarwal G (2005). The genome sequence of Trypanosoma cruzi, etiologic agent of Chagas disease.. Science.

[34] Weatherly DB, Boehlke C, Tarleton RL (2009). Chromosome level assembly of the hybrid Trypanosoma cruzi genome.. BMC Genomics.

[35] Ivens AC, Peacock CS, Worthey EA, Murphy L, Aggarwal G (2005). The genome of the kinetoplastid parasite, Leishmania major.. Science.

[36] Parsons M, Worthey EA, Ward PN, Mottram JC (2005). Comparative analysis of the kinomes of three pathogenic trypanosomatids: Leishmania major, Trypanosoma brucei and Trypanosoma cruzi.. BMC Genomics.

[37] Ward P, Equinet L, Packer J, Doerig C (2004). Protein kinases of the human malaria parasite Plasmodium falciparum: the kinome of a divergent eukaryote.. BMC Genomics.

[38] Manning G, Whyte DB, Martinez R, Hunter T, Sudarsanam S (2002). The protein kinase complement of the human genome.. Science.

[39] Daub H, Olsen JV, Bairlein M, Gnad F, Oppermann FS (2008). Kinase-selective enrichment enables quantitative phosphoproteomics of the kinome across the cell cycle.. Mol Cell.

[40] Nolen B, Taylor S, Ghosh G (2004). Regulation of protein kinases; controlling activity through activation segment conformation.. Mol Cell.

[41] Wiese M, Kuhn D, Grunfelder CG (2003). Protein kinase involved in flagellar-length control.. Eukaryot Cell.

[42] Erdmann M, Scholz A, Melzer IM, Schmetz C, Wiese M (2006). Interacting protein kinases involved in the regulation of flagellar length.. Mol Biol Cell.

[43] Pradel LC, Bonhivers M, Landrein N, Robinson DR (2006). NIMA-related kinase TbNRKC is involved in basal body separation in Trypanosoma brucei.. J Cell Sci.

[44] Schwartz D, Gygi SP (2005). An iterative statistical approach to the identification of protein phosphorylation motifs from large-scale data sets.. Nat Biotechnol.

[45] Conesa A, Gotz S, Garcia-Gomez JM, Terol J, Talon M (2005). Blast2GO: a universal tool for annotation, visualization and analysis in functional genomics research.. Bioinformatics.

[46] Contreras VT, Araujo-Jorge TC, Bonaldo MC, Thomaz N, Barbosa HS (1988). Biological aspects of the Dm 28c clone of Trypanosoma cruzi after metacyclogenesis in chemically defined media.. Mem Inst Oswaldo Cruz.

[47] Camargo EP (1964). Growth and Differentiation in Trypanosoma Cruzi. I. Origin of Metacyclic Trypanosomes in Liquid Media.. Rev Inst Med Trop Sao Paulo.

[48] de Sousa MA (1983). A simple method to purify biologically and antigenically preserved bloodstream trypomastigotes of Trypanosoma cruzi using DEAE-cellulose columns.. Mem Inst Oswaldo Cruz.

[49] Larsen MR, Thingholm TE, Jensen ON, Roepstorff P, Jorgensen TJ (2005). Highly selective enrichment of phosphorylated peptides from peptide mixtures using titanium dioxide microcolumns.. Mol Cell Proteomics.

[50] Pinkse MW, Uitto PM, Hilhorst MJ, Ooms B, Heck AJ (2004). Selective isolation at the femtomole level of phosphopeptides from proteolytic digests using 2D-NanoLC-ESI-MS/MS and titanium oxide precolumns.. Anal Chem.

[51] Rappsilber J, Ishihama Y, Mann M (2003). Stop and go extraction tips for matrix-assisted laser desorption/ionization, nanoelectrospray, and LC/MS sample pretreatment in proteomics.. Anal Chem.

[52] Gnad F, Ren S, Cox J, Olsen JV, Macek B (2007). PHOSIDA (phosphorylation site database): management, structural and evolutionary investigation, and prediction of phosphosites.. Genome Biol.

