# Supplementary figures and images for: Profiling the Trypanosoma cruzi Phosphoproteome
Source: PLoS One. 2011 Sep 22;6(9):e25381. doi: 10.1371/journal.pone.0025381 (PMC3178638; doi:10.1371/journal.pone.0025381)

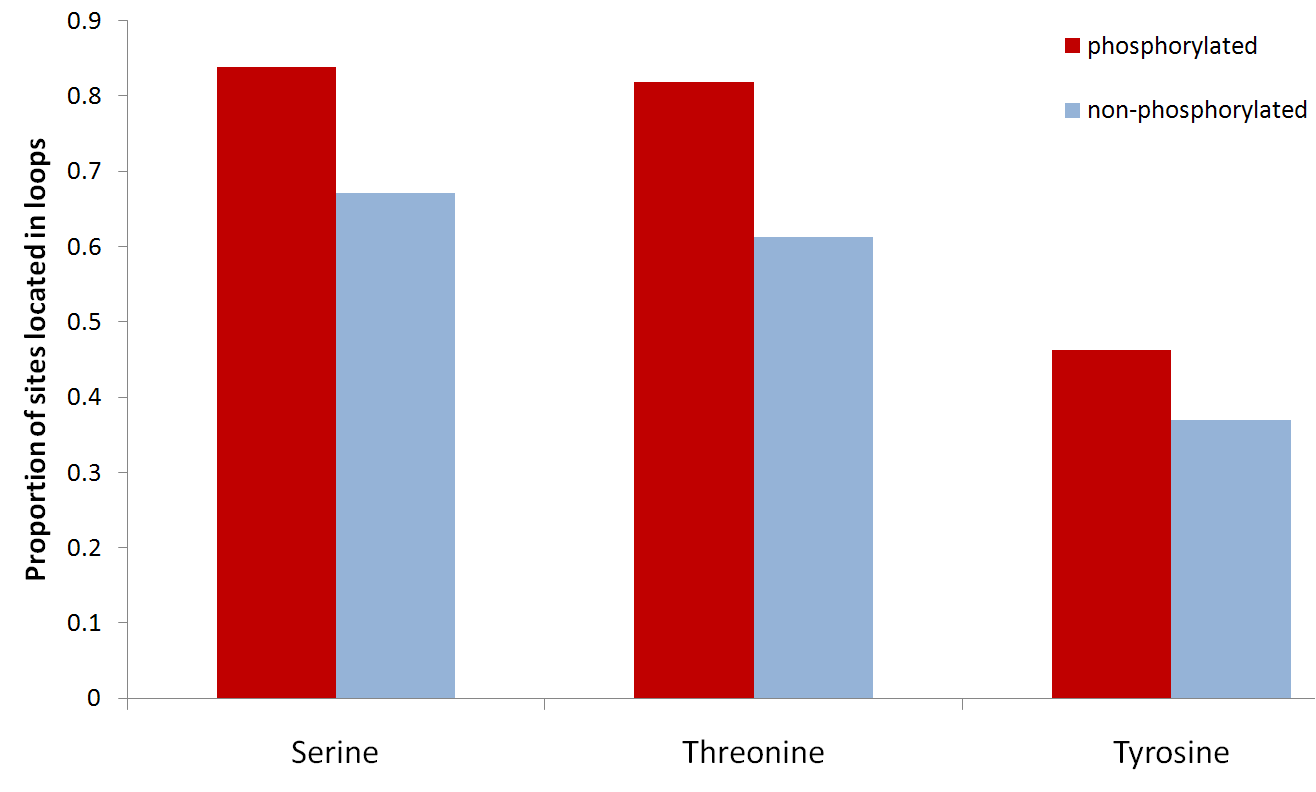

Supplement: Figure S1 — Proportion of residues located in loops. Proportion of phosphorylated (red) and non-phosphorylated (blue) serines, threonines and tyrosines that are located in loops, according to secondary structure prediction. The phosphorylated residues are significantly higher localized in loops and turns when compared with their non-phosphorylated counterparts. (TIFF) [file pone.0025381.s001.tiff]

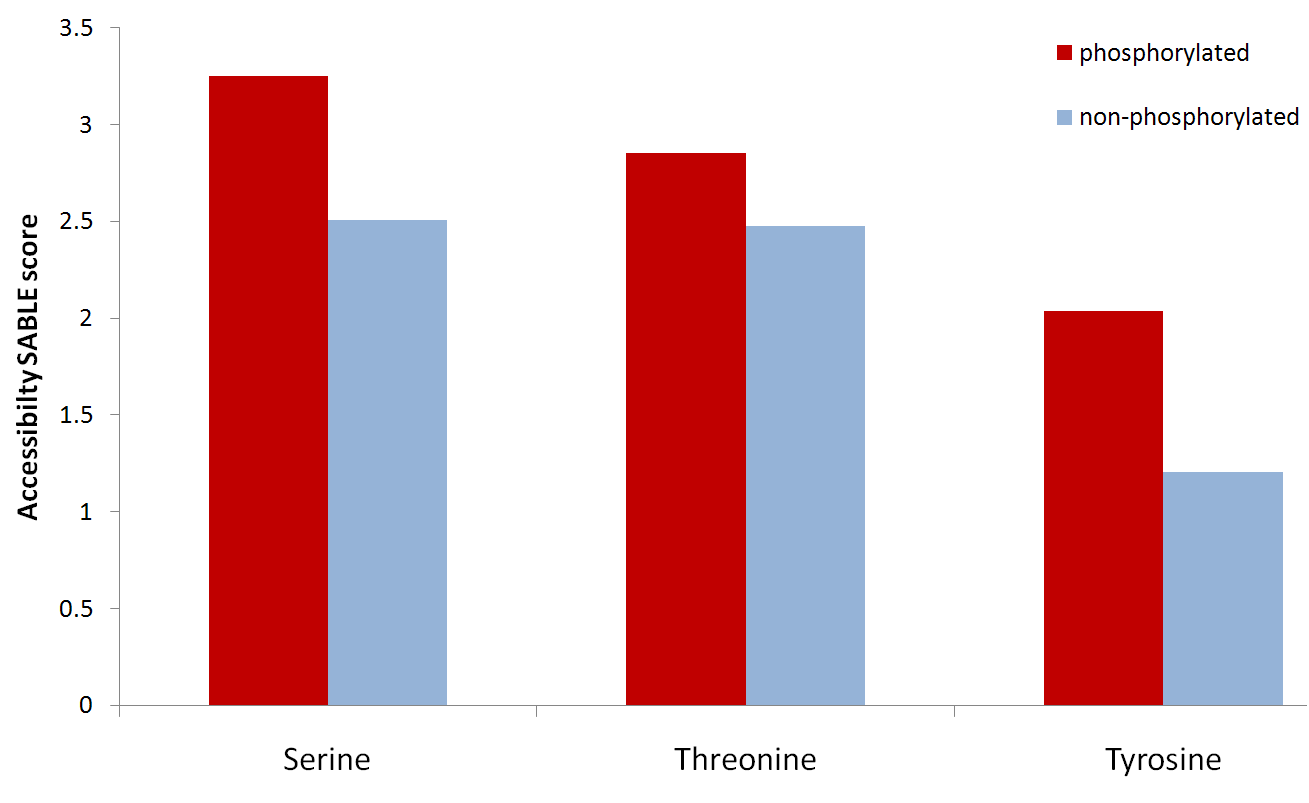

Supplement: Figure S2 — S/T/Y average accessibility. Average accessibility of phosphorylated (red) and non-phosphorylated (blue) serines and threonines according to secondary structure prediction. The predicted average accessibilities of T. cruzi phosphorylation sites were found to be significantly higher than the accessibilities of non-phosphorylated sites. (TIFF) [file pone.0025381.s002.tif]

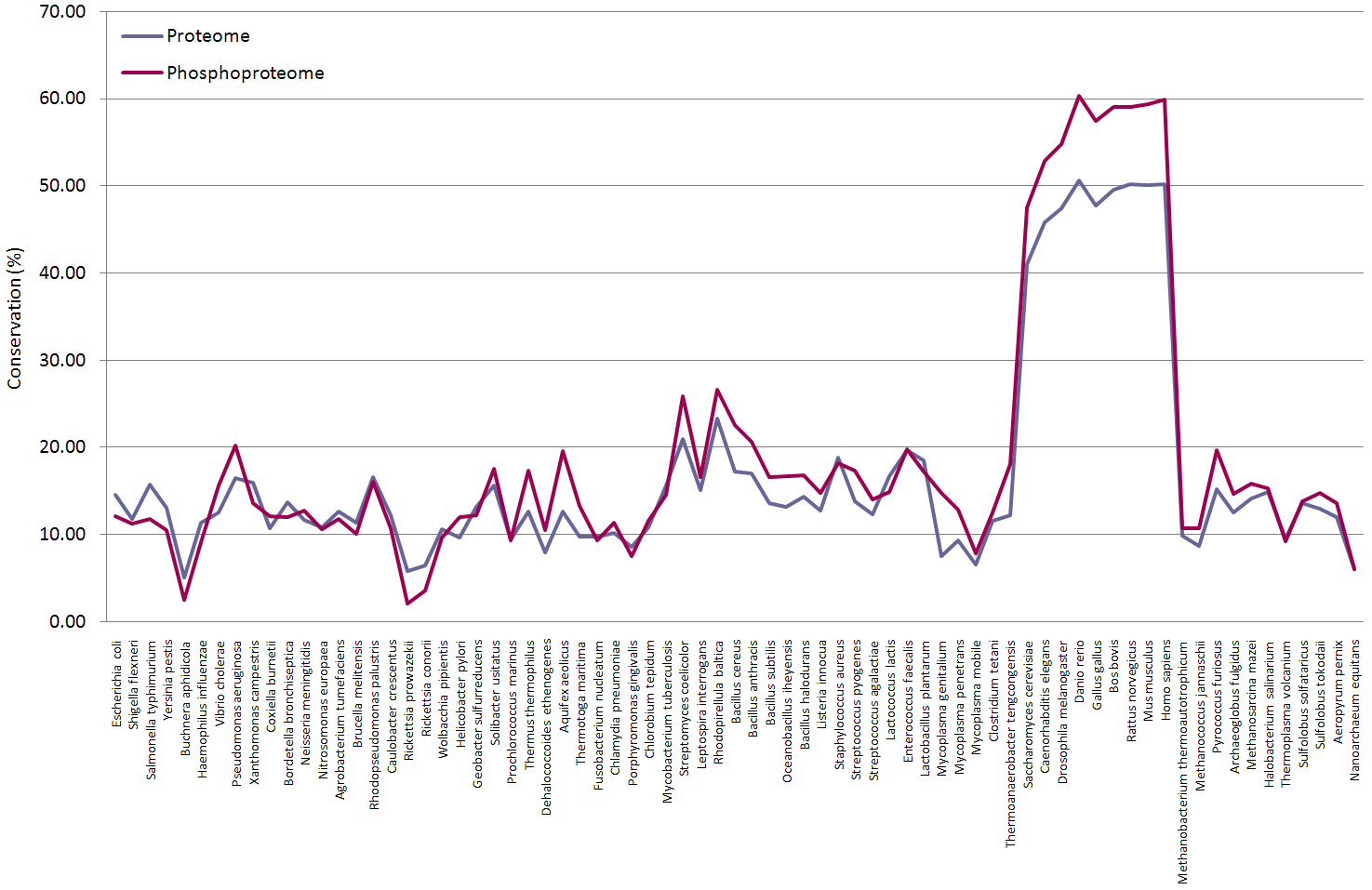

Supplement: Figure S3 — Phosphoproteome similarity on all domains of life. Homologs of all T. cruzi proteins were derived and the conservation of non-phosphorylated (blue) and phosphorylated (red) proteins were accessed across a range of both prokaryotic and eukaryotic organisms. When compared to other eukaryotic species, the percentage of T. cruzi phosphorylated proteins that have homologs is significantly higher than that of the non-phosphorylated proteins. On the other hand, in the prokaryotic domain, phosphorylated T. cruzi proteins are as conserved as the non-phosphorylated ones. (TIFF) [file pone.0025381.s003.tiff]

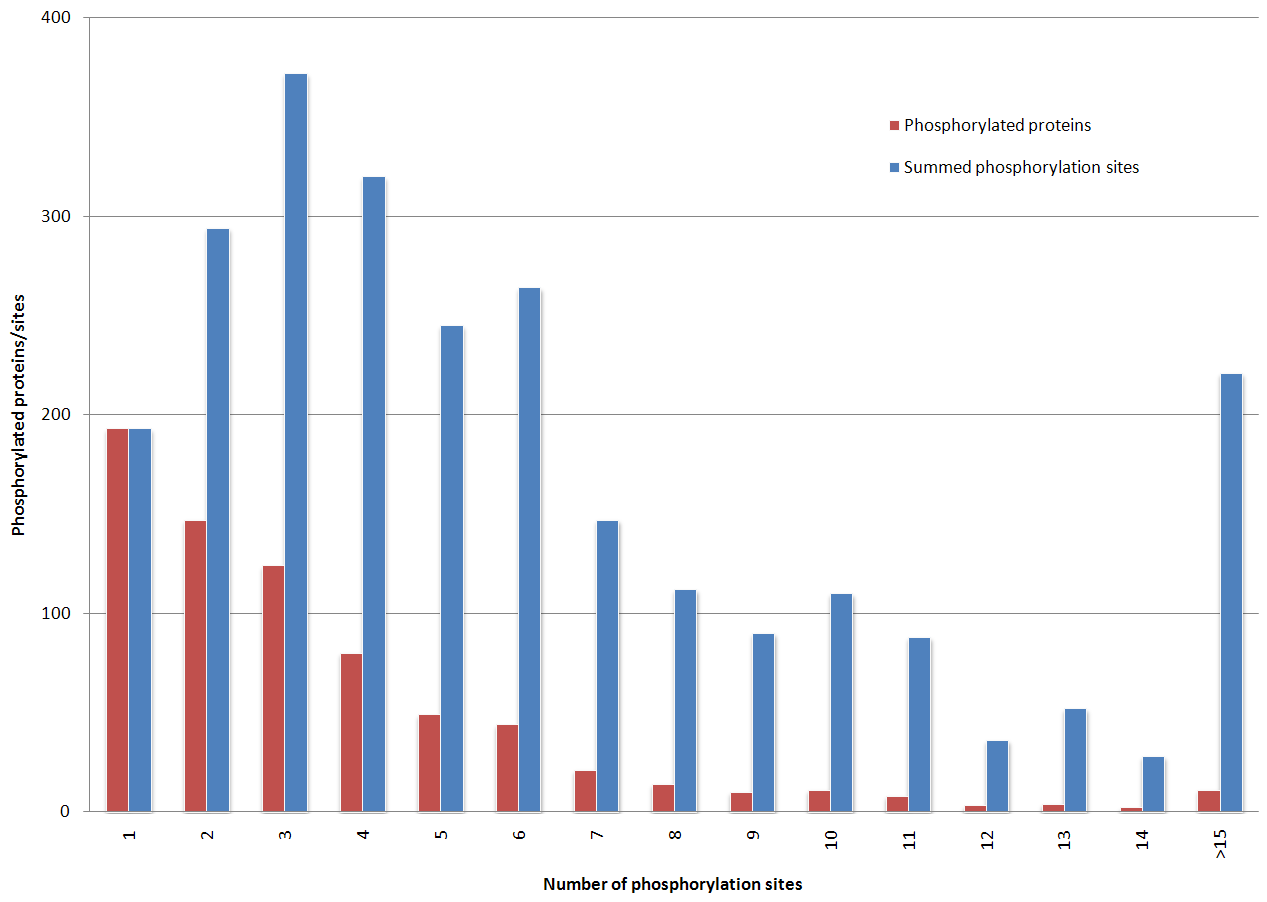

Supplement: Figure S4 — Protein phosphorylation distribution. Proteins were binned according to their number of phosphorylation sites, as determined by LC-MS/MS. Bars represent the number of proteins belonging to each category bin (red) and the summed number of phosphorylation sites derived from the proteins in each respective class (blue). (TIFF) [file pone.0025381.s004.tiff]

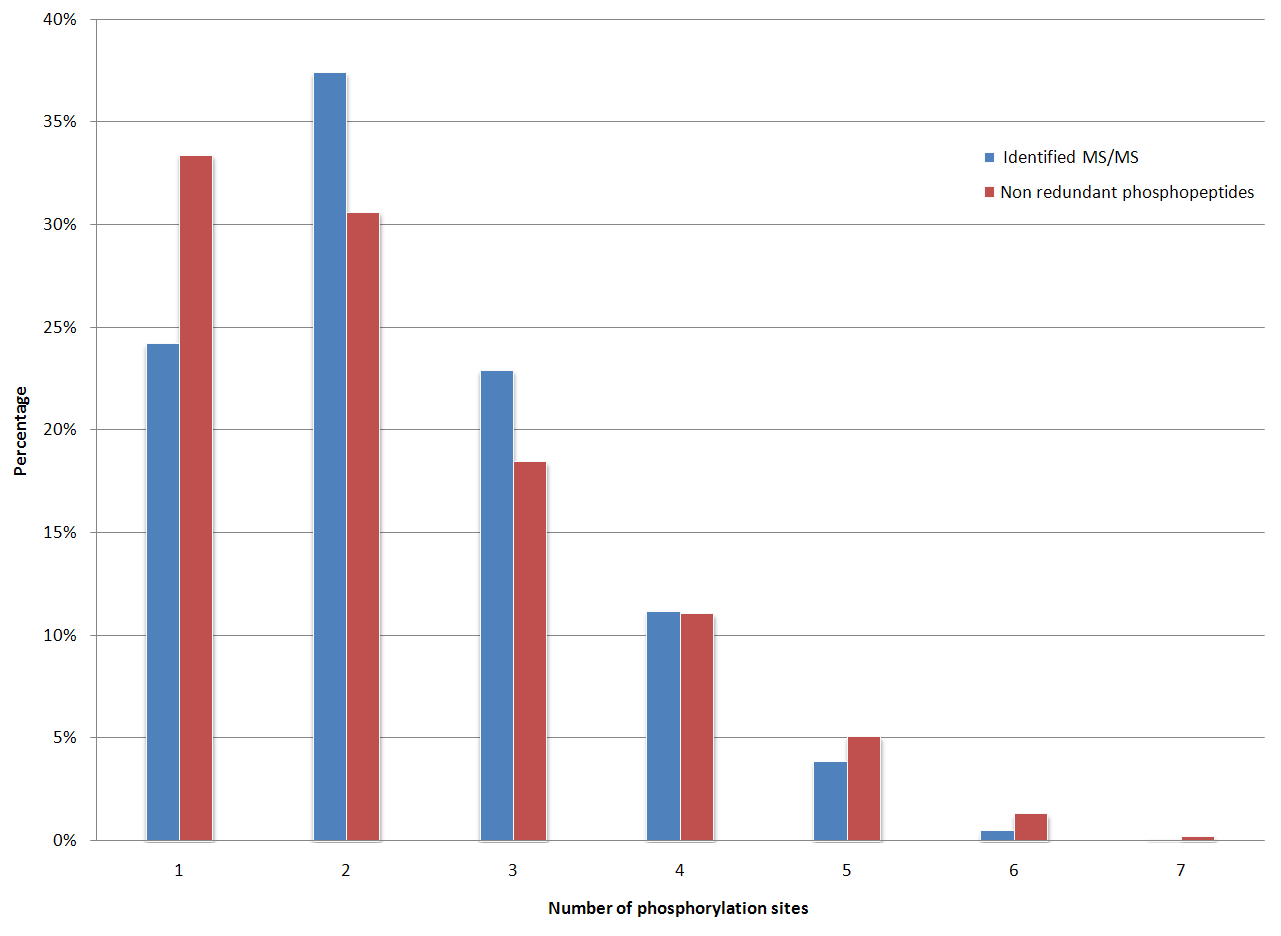

Supplement: Figure S5 — Peptide phosphorylation distribution. Peptides were binned according to their number of phosphorylation sites, as determined by LC-MS/MS. Bars represent the number of non-redundant phosphorylated peptides belonging to each category bin (red) and the total number of MS/MS spectra identifying the phosphopeptides of the corresponding bin (blue). (TIFF) [file pone.0025381.s005.tiff]
